# Organization of projection from brainstem auditory nuclei to the inferior colliculus of Japanese house bat (*Pipistrellus abramus*)

**DOI:** 10.1002/brb3.1059

**Published:** 2018-07-12

**Authors:** Tetsufumi Ito, Takafumi Furuyama, Kazuma Hase, Kohta I. Kobayasi, Shizuko Hiryu

**Affiliations:** ^1^ Department of Anatomy Kanazawa Medical University Uchinada Japan; ^2^ Research and Education Program for Life Science University of Fukui Fukui Japan; ^3^ Neuroethology and Bioengineering Laboratory Faculty of Life and Medical Sciences Doshisha University Kyotanabe Japan

**Keywords:** inferior colliculus, large GABAergic neurons, subcortical auditory pathways, synaptic domain

## Abstract

**Objectives:**

Echolocating bats show remarkable specialization which is related to analysis of echoes of biosonars in subcortical auditory brainstem pathways. The inferior colliculus (IC) receives inputs from all lower brainstem auditory nuclei, i.e., cochlear nuclei, nuclei of the lateral lemniscus, and superior olivary complex, and create de novo responses to sound, which is considered crucial for echolocation. Inside the central nucleus of the IC (ICC), small domains which receive specific combination of extrinsic inputs are the basis of integration of sound information. In addition to extrinsic inputs, each domain is interconnected by local IC neurons but the cell types related to the interconnection are not well‐understood. The primary objective of the current study is to examine whether the ascending inputs are reorganized and terminate in microdomains inside the ICC.

**Methods:**

We made injection of a retrograde tracer into different parts of the ICC, and analyzed distribution of retrogradely labeled cells in the auditory brainstem of Japanese house bat (*Pipistrellus abramus*).

**Results:**

Pattern of ascending projections from brainstem nuclei was similar to other bat species. Percentages of labeled cells in several nuclei were correlated each other. Furthermore, within the IC, we identified that large GABAergic (LG) and glutamatergic neurons made long‐range connection.

**Conclusions:**

Synaptic organization of IC of Japanese house bat shows specialization which is likely to relate for echolocation. Input nuclei to the IC make clusters which terminate in specific part of the ICC, implying the presence of microdomains. LG neurons have roles for binding IC microdomains.

## 1 INTRODUCTION

Echolocating bats emit sound pulses with specific acoustical features to targets, and measure the distance to the target according to the echoes of the sound pulses. To analyze the echoes and pulses themselves, auditory nuclei of bats are remarkably hypertrophied (Zook & Casseday, 1982a), and show specialization related to echolocation (Covey & Casseday, 1995), which is obvious in the medial superior olive (MSO) of the superior olivary complex (SOC) (Casseday, Covey, & Vater, 1988; Covey, Vater, & Casseday, 1991; Grothe, Park, & Schuller, 1997; Grothe, Schweizer, Pollak, Schuller, & Rosemann, 1994) and monaural nuclei of the lateral lemniscus, namely intermediate nucleus (ILL) and compact and multipolar cell divisions of ventral nucleus (VLLc and VLLm) (Covey & Casseday, 1991). Lower brainstem nuclei, namely, cochlear nuclei (CN), SOC, and nuclei of the lateral lemniscus (NLL), analyze specific features of sound and send the analyzed information to the inferior colliculus (IC), which is the largest nuclei in entire subcortical auditory system (Ito, Bishop, & Oliver, 2016). Since all the sound information is converged, the IC is considered to integrate sound information and create coding of complex sound information: In bats, analyses related to echolocation such as tunings of sound duration (Casseday, Ehrlich, & Covey, 1994), and delay coding (Portfors & Wenstrup, 1999) are found in the IC.

It has been proposed that IC local circuit is composed of multiple small domains, i.e., “synaptic domains”, which receive specific combination of extrinsic inputs (Cant & Benson, 2006; Casseday, Fremouw, & Covey, 2002; Loftus, Bishop, & Oliver, 2010; Oliver, 2005), and contain three cell types, i.e., large GABAergic (LG), small GABAergic (SG), and glutamatergic (GLU) cells, that have different input and output patterns (Ito & Oliver, 2012). Neurons in a synaptic domain likely to make influence on different synaptic domains since most IC neurons shown to have local collaterals (Oliver, Kuwada, Yin, Haberly, & Henkel, 1991; Wallace, Shackleton, & Palmer, 2012), although the knowledge about cell types that influence on other synaptic domains is poorly known. As the three cell types organize different patterns of neuronal connection, we hypothesized that specific cell types are involved in connecting different synaptic domains in the IC.

In this study, we made injection of fluorogold (FG), a retrograde tracer, into different parts of the ICC, and analyzed distribution of retrogradely labeled cells in the entire auditory brainstem pathways of Japanese house bat (*Pipistrellus abramus*), which uses frequency modulated (FM) pulses for echolocation, to examine whether the ascending inputs are reorganized and terminate in small domains inside the ICC. Furthermore, by analyzing the distribution of retrogradely labeled cells inside the IC, we identified cell types that make long‐range intrinsic connection inside the IC. Although this species have been used frequently on biosonar studies (Fujioka et al., 2014; Sumiya, Fujioka, Motoi, Kondo, & Hiryu, 2017; Takahashi et al., 2014; Yamada, Hiryu, & Watanabe, 2016), the information about the organization of auditory pathways of this species has not been reported except for a single unit recording study from the IC (Goto, Hiryu, & Riquimaroux, 2010). Therefore, our study will give new neuroethological insights on biosonar.

## 2 MATERIALS AND METHODS

### 2.1 Subjects

Japanese house bats (*Pipistrellus abramus*) were used in this study. *P. abramus* is a member of the *Vespertilionidae* and is commonly found in Japan. The bats were captured from a large colony roosting on bridge girders near the campus of Doshisha University, Kyotanabe, Japan. The bats were kept in a humidity‐ and temperature‐controlled rearing cage, and were allowed free access to food (mealworms) and water. Experiments were conducted on seven adult bats of both sexes. All experiments were conducted in accordance with institutional guidelines at the University of Fukui and the Doshisha University. All efforts were made to minimize the number of animals used and their suffering.

### 2.2 Surgery

Bats were anesthetized with isoflurane mixed with oxygen for the duration of the surgery. Skin covering the skull was cut at the midline. A small screw was fixed at Bregma with instant glue, and the animal head was fixed by holding the screw. A craniotomy opened the right parietal bone to expose the IC. Glass micropipettes (4–7 μm tip) were filled with 3% FG (Fluorochrome, Denver, CO, USA) diluted in saline. FG was iontophoretically injected from the recording pipette with a 2–5 μA positive current for durations of 2–5 min with a 50% duty cycle (7 s on/7 s off) using a constant current injector (BCC‐3621; Bio‐Medica, Osaka, Japan). Twenty‐four hours after the surgery, the bats were deeply anesthetized with pentobarbital sodium (64.8 mg/kg, i.p.) and perfused transcardially with 4% paraformaldehyde in 0.1 M phosphate buffer (pH 7.4). The brains were dissected out, and immersed in the same fixative overnight. After cryoprotection with 30% sucrose in 0.1 M phosphate buffer (pH 7.4) at 4°C for 1–2 days, brains were cut at the level of superior colliculus (SC), and 30 μm‐thick serial coronal sections of the brainstem were cut with a freezing microtome. Every 5th section was used for histology. Three sets of every 5th section was used and processed for Nissl staining, bright‐field immunostaining for FG, and fluorescent immunostaining for FG, glutamic acid decarboxylase 67 (GAD67), and vesicular glutamate transporter 2 (VGLUT2). We found that the angle of sectioning differed among animals, and we estimated the angle from the 3D reconstruction of the brainstem (shown in top of Figures 3, 4, 5).

### 2.3 Antibodies

In this study, mouse monoclonal antibody for GAD67 (antiserum MAB5406; Merck, Darmstadt, Germany, http://scicrunch.org/resolver/AB_2278725) and rabbit polyclonal antibodies for VGLUT2 (Fujiyama, Furuta, & Kaneko, 2001) and FG (AB153; Merck, http://scicrunch.org/resolver/AB_2632408) were used. Specificity of the antibody for FG was confirmed by absence of staining in bat brain which did not receive injection of FG. The immunogen used for anti‐GAD67 antibody was a recombinant whole protein of rat GAD67. We tested homology of rat GAD67 by using Protein BLAST (https://blast.ncbi.nlm.nih.gov/), and found that the amino acid sequence of rat GAD67 protein (Accession No: NP_058703.1) shared 96% identity to predicted sequence of GAD67 of big brown bat (*Eptesicus fuscus*) (Accession: XP_008136804.1), which is another member of the *Vespertilionidae*. In a previous study (Ito et al., 2007), we confirmed the specificity of the anti‐GAD67 by western blot and an absorption test. In the western blot, a single band around 67 kDa was detected (which is also described in manufacturer's sheet). No signal was detected in rat brain sections incubated with the antibody (1:3,000) preabsorbed with recombinant rat GAD67 protein (180 μg/ml). Furthermore, the staining pattern of the antibody was consistent with that in a previous study (Vater, Kossl, & Horn, 1992).

The immunogen used for anti‐VGLUT2 antibody was a synthetic peptide to C‐terminal amino acid residues CWPNGWEKKEEFVQESAQDAYSYKDRDDYS of rat VGLUT2 (aa. 554–582), which has 79% similarity in Protein BLAST to the corresponding part of predicted sequence of VGLUT2 of big brown bat (Accession: XP_008147186.1). Specificity for the antibody was characterized by western blot and an absorption test (Fujiyama et al., 2001). In the western blot, a single band around 62 kDa was detected. No signal was detected in rat brain sections incubated with antibody preabsorbed with 10,000‐fold (in mol) excess amounts of the synthetic peptide used for the immunization.

### 2.4 Immunohistochemistry

For bright‐field microscopy, sections were incubated with the rabbit anti‐FG antibody (1:8,000) diluted in 0.3% Triton X‐100 with 1% normal goat serum in 0.05 M phosphate‐buffered saline (PBS‐XG), followed by an incubation in a biotinylated goat anti‐rabbit secondary antibody (1:200; Vector Laboratories, Burlingame, CA, USA) diluted in PBS‐XG. After an incubation in avidin‐biotinylated peroxidase complex (1:50; ABC‐Elite, Vector) in PBS containing 0.3% Triton X‐100, sections were processed for a Nickel‐diaminobenzidine reaction. Sections were mounted on coated glass slides. In some cases, sections were counterstained with Neutral Red (Fisher Scientific, Fair Lawn, NJ, USA). Sections were dehydrated through graded alcohols, cleared with xylene, and coverslipped with Entellan (Merck).

For fluorescent microscopy, sections were incubated in 0.01 M sodium citrate (pH 6.0) at 80°C for 2 hr to enhance immunoreactivity for GAD67 and VGLUT2. After a brief wash, the sections were incubated overnight with guinea pig anti‐VGLUT2 (0.5 μg/ml), mouse anti‐GAD67 (1:1,000), and rabbit anti‐FG (1:2,000) diluted in PBS‐XG. The next day, sections were washed and incubated with donkey AlexaFluor 488‐conjugated anti‐rabbit IgG, donkey Cy3‐conjugated anti‐guinea‐pig IgG, and donkey Cy5‐conjugated anti‐mouse IgG (1:200, Jackson Immunoresearch, West Grove, PA, USA). Sections were mounted on coated slides, air dried, rehydrated, and coverslipped with 1,4‐diazabicyclo[2.2.2]octane.

### 2.5 Imaging

Bright field micrographs were acquired with an upright microscope (AX80; Olympus, Tokyo, Japan) equipped with ×4 and ×10 lenses (NA = 0.16 and 0.4, respectively) and a digital camera (DMC‐GH3; Panasonic, Osaka, Japan). To compensate non‐uniform illumination, a blank image was acquired, and subtracted from original microscopic images.

Fluorescent images of entire IC were acquired with a laser scanning confocal microscope (Zeiss 710; Carl Zeiss Microimaging, Germany) and a ×63 lens (N.A. = 1.4). AlexaFluor 488 was excited by a 488 nm Ar laser, and the emitted fluorescence was filtered with a 500–530 nm bandpass filter. Cy3 was excited by a 543 nm He–Ne laser, and the emitted fluorescence was filtered with a 565–615 nm bandpass filter. Cy5 was excited by a 633 nm He–Ne laser, and emitted fluorescence was filtered with a 650 nm low‐pass filter. Images of each dye were taken sequentially to avoid bleed‐through artifact. Tiled images of IC was obtained and montaged with a custom script on MATLAB (Mathworks, Natick, MA, USA). Minimal adjustments of the fluorescence intensity levels were made in Photoshop CS3 (Adobe Systems, San Jose, CA, USA).

### 2.6 Data analysis

We plotted the FG‐positive (+) cells in the entire auditory brainstem nuclei using a bright‐field microscope (BX50; Olympus) equipped with a motorized stage (Bioprecision, Ludl Electronic Products, Hawthorne, NY, USA) that is controlled by Neurolucida (MBF Bioscience, Williston, VT, USA). Fluorescent montage images of the IC were imported to Neurolucida and the distribution of FG+ GABAergic neurons were analyzed. Injection sites were defined as the location where the FG immunoreactivity was present in extracellular space, and the center of injection was defined as the site of lesion caused by the current injection. The boundary of auditory nuclei was identified by cytoarchitecture revealed by Nissl counterstain or differential interference contrast optics. The Neurolucida traces were exported and further analyzed with a custom‐made script working on MATLAB. Shrinkage correction was not performed.

To make quantitative analysis of distribution of FG+ GABAergic and non‐GABAergic neurons around the injection site and ipsilateral IC, distance from the center of injection was calculated for every cell, and histograms of the distance with a bin width of 100 μm were created. In each bin, the ratio of SG and LG neurons to total labeled cells was calculated, and the relationship between distance and ratio was compared between LG and SG cells using 2‐way analysis of variance (ANOVA).

## 3 RESULTS

### 3.1 Sites of injection

**Figure 1:**
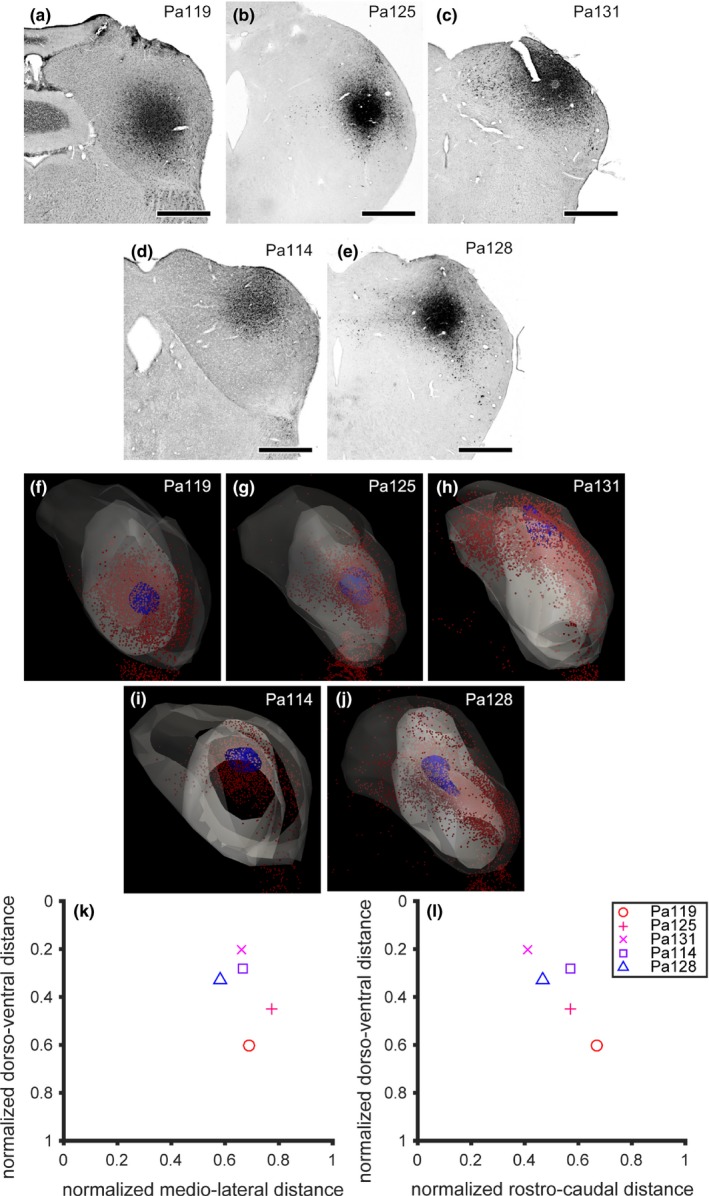
Injection sites of fluorogold (FG). (a–e) Bright‐field micrographs of the inferior colliculus (IC) showing the center of injection. Note that sections of Pa119 and Pa131 were counterstained with Neutral Red. Scale bars: 500 μm. (f–j) Three‐dimensional reconstruction of ipsilateral IC. Dark regions indicate the outlines of IC, lighter regions indicate the central nucleus (ICC), and blue regions indicate the center of injection. Red dots indicates FG‐labeled cell bodies. According to the injection sites and distribution of cell bodies, we determined that the injection was made in ventro‐lateral‐posterior, dorso‐lateral, dorso‐intermediate‐anterior, dorso‐intermediate, and dorso‐medial part of the ICC of Pa119, Pa125, Pa131, Pa114, and Pa128 respectively. (k, l) Normalized location of injection sites. Medio‐lateral, dorso‐ventral, and rostro‐caudal positions of injection sites were normalized by the medio‐lateral, dorso‐ventral, and rostro‐caudal length of the IC. Symbols indicate individual cases

In all seven cases, the injection of FG was made inside the IC, and we analyzed the distribution of FG+ in the local circuit of the hemilateral IC that received injection (referred as “ipsilateral IC” in the following). Since two cases showed leakage of FG injection to the periaqueductal gray, we excluded the cases from the analysis of ascending input to the IC from lower auditory brainstem nuclei. Remaining five cases showed no leakage of FG injection outside the IC (Figure 1a–e), and we analyzed these cases for further analysis about the ascending projection to the IC. Three‐dimensional reconstruction of the ipsilateral IC (dark gray area in Figure 1f–j) revealed that the injection site (blue area), which is defined as the diffusion of FG in the extracellular space, was restricted in the central nucleus of the IC (ICC; light gray area) in 4 cases (Pa119, Pa125, Pa114, and Pa128). In the ipsilateral IC of these cases, majority of FG+ IC cell bodies (red dots in Figure 1f–j) was found in the ICC. In one case (Pa131), injection was made in the boundary of the ICC and part of lateral cortex that lied dorsal to the ICC, and many FG+ IC cells were found in the cortical areas of the IC.

In Pa119, of which injection was made large, FG+ cells were distributed in the ventral half of the ICC and arranged spherically (Figure 1f). The “sphere” of FG+ cells located laterally in the ICC. In the other cases, FG+ cells were distributed more dorsally, and arranged in more flattened region inside the ICC. In Pa125, FG+ cells were mainly distributed in the lateral half of the ICC (Figure 1g), while in Pa128 more FG+ cells were found in the medial part of the ICC (Figure 1j). Medio‐lateral location of injection in Pa131 and Pa114 was determined as intermediate because the center of injection was located medially while FG+ cells were distributed widely in the medio‐lateral axis (Figure 1h,j).

To obtain objective classification of injection sites, we normalized the location of injection sites, since amount of shrinkage during histology seemed to be different among cases. We measured medio‐lateral distance between medial edge of the IC and injection center and distance between medial and lateral edges of the IC, and calculated the medio‐lateral ratio between them. The medio‐lateral ratios of Pa119, Pa125, Pa131, Pa114, and Pa 128 were 0.691, 0.772, 0.662, 0.665, and 0.580 respectively. Since the angle of sectioning is different among cases (top drawings in Figures 3–5), we used three‐dimensional reconstruction to measure dorso‐medial and rostro‐caudal ratios. After the 3D models were rotated, ratio of distance between dorsal surface of the IC and injection center to distance between dorsal surface and ventral edge of the IC was calculated as dorso‐ventral ratio. The dorso‐ventral ratios of Pa119, Pa125, Pa131, Pa114, and Pa 128 were 0.600, 0.450, 0.203, 0.282, and 0.331, respectively. In a single unit recording study from Japanese house bat IC (Goto et al., 2010), dorso‐ventral length of the IC was around 2.0 mm, and region shallower than 1.0 mm (dorso‐ventral ratio <0.5) showed best frequency lower than 40 kHz, which is the quasi constant frequency of the sonar pulse, and region deeper than 1.0 mm (dorso‐ventral ratio >0.5) did not contain unit with best frequency lower than 40 kHz. Therefore, we assigned Pa119 as high frequency injection case, and other cases as low frequency injection cases. To calculate rostro‐caudal ratio, distance between rostral end of the IC to the injection center was divided by distance between rostral and caudal ends of the IC. The rostro‐caudal ratios of Pa119, Pa125, Pa131, Pa114, and Pa 128 were 0.667, 0.571, 0.412, 0.571, and 0.467, respectively.

Based upon the location of injection site and distribution of FG+ cells inside the ICC (Figure 1k,l), we classified the location of injection of Pa119 as ventro‐lateral‐posterior, that of Pa125 as dorso‐lateral, that of Pa131 as dorso‐intermediate‐anterior, that of Pa114 as dorso‐intermediate, and that of Pa128 as dorso‐medial.

Labeled cells that were found close to the injection site were likely to take up the tracer directly from the dendrites and cell bodies as well as axonal terminals, and therefore we cannot distinguish retrogradely labeled cells and direct labeled cells. On the other hand, labeled cells of which distal end of dendritic trees was outside the diffusion of tracer are considered as retrogradely labeled cells. In all the seven cases, the radius of tracer diffusion was ranged from 88.1 to 177.9 μm. In Japanese house bats, the maximal length of a dendritic tree was approximately 170 μm, which was measured from Golgi‐stained materials (not shown). In rats, the maximal width of dendritic arborization is smaller than 400 μm (Malmierca, Blackstad, Osen, Karagulle, & Molowny, 1993). Therefore, if the cell bodies of labeled neurons were more than 200 μm distant from the edge of tracer diffusion, they are regarded as retrogradely labeled cells. Finally, in the current samples, labeled cells which were more than 400 μm distant from the center of the injection were regarded as retrogradely labeled.

### 3.2 Distribution of retrogradely labeled cells in brainstem auditory nuclei

**Figure 2:**
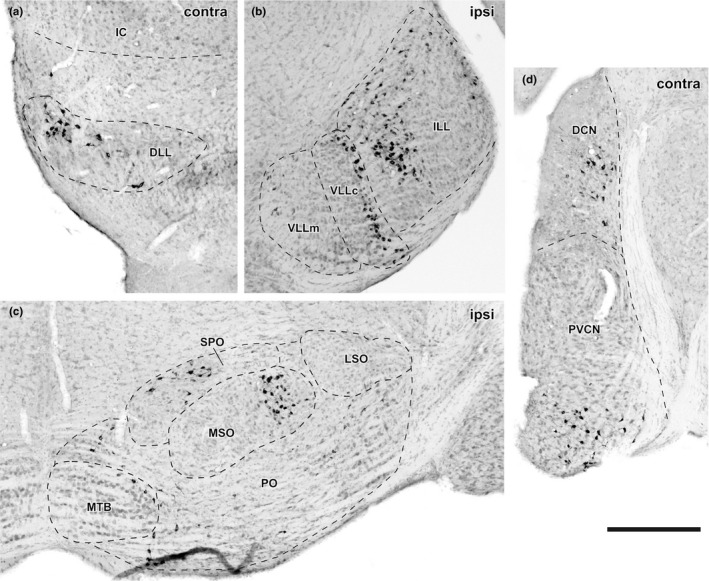
Bright‐field micrographs of Pa131, of which injection were made in dorsal ICC, showing retrogradely labeled cells in contralateral dorsal nucleus of the lateral lemniscus (DLL) (a), ipsilateral nuclei of the lateral lemniscus (NLL) (b), ipsilateral superior olivary complex (SOC) (c), and contralateral cochlear nuclei (CN) (d). (a) In the DLL, retrogradely labeled cells were found in the lateral part. (b) In the intermediate nucleus (ILL), labeled cells were found in the periphery of the nucleus. In the compact cell division of the ventral nucleus (VLLc), labeled cells were found latero‐dorsally, while in the multipolar cell division (VLLm), labeled cells were found medially. (c) SOC was subdivided into medial superior olive (MSO), lateral superior olive (LSO), superior paraolivary nucleus (SPO), medial nucleus of the trapezoid body (MTB), and periolivary nuclei (PO). Retrogradely labeled cells were densely distributed in the MSO and SPO. In the SPO, the fragments of labeled dendrites were seen in the lateral part of the nucleus. Note: retrogradely labeled cells in the periphery of the MTB. (d) In the dorsal nucleus (DCN), labeled cells were found in ventral part of deep layers. In the posteroventral nucleus (PVCN), labeled cells were concentrated in the ventral part. Sections were counterstained with Neutral Red. Scale bar indicates 300 μm

**Figure 3:**
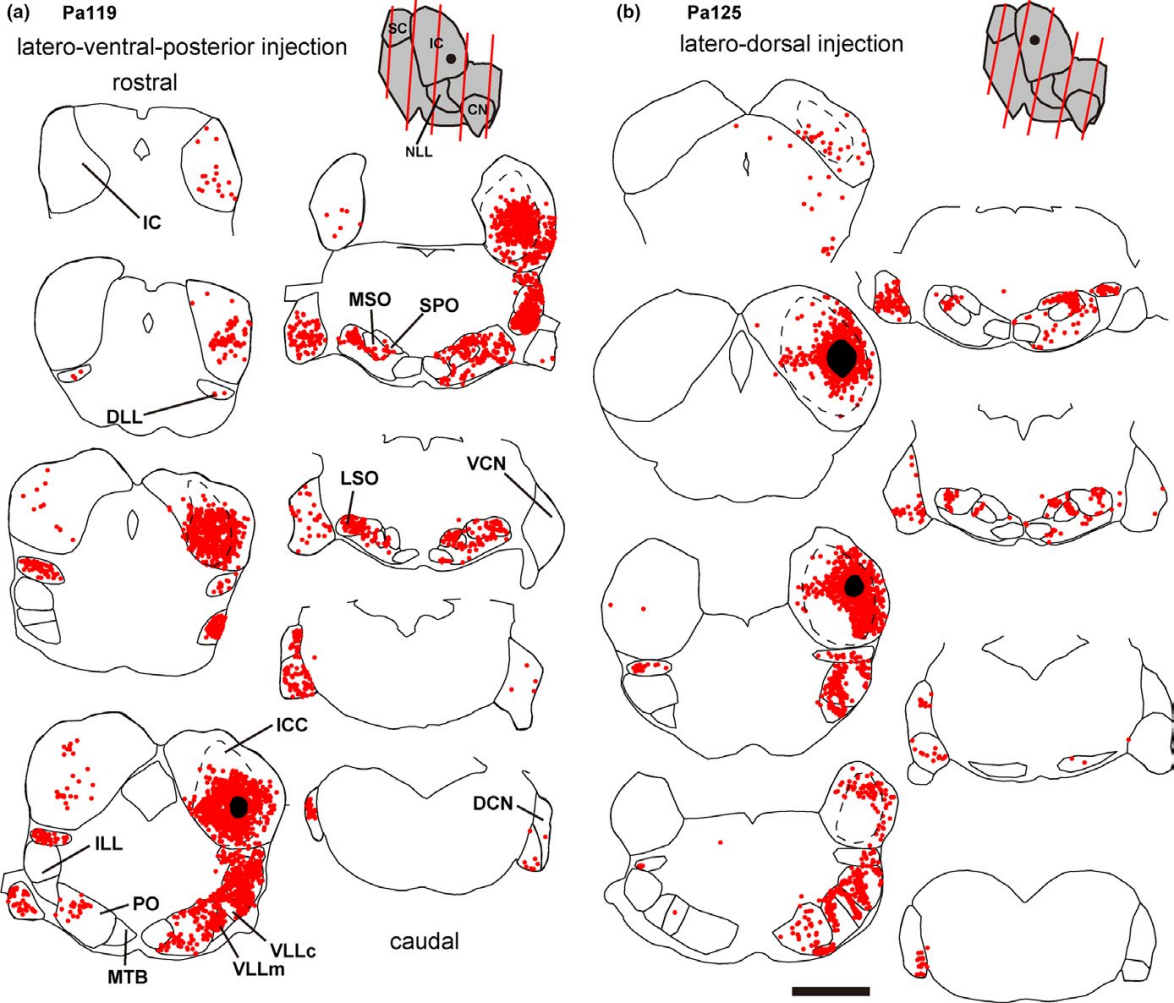
Line drawing showing the traces of auditory nuclei and distribution of labeled cell bodies (red dots). Black filled area indicate the injection site, and dotted lines indicate the border of ICC. Drawings filled with gray indicate side view of brainstem,plane of sectioning (red lines), and injection site (a black circle), which was determined from 3D reconstruction. (a) Pa119, of which large injection was made in the ventro‐lateral ICC. (b) Pa125, a dorso‐lateral injection case. Sections were represented from rostral to caudal, and each section was separated 300 μm. Scale bar: 1 mm

**Figure 4:**
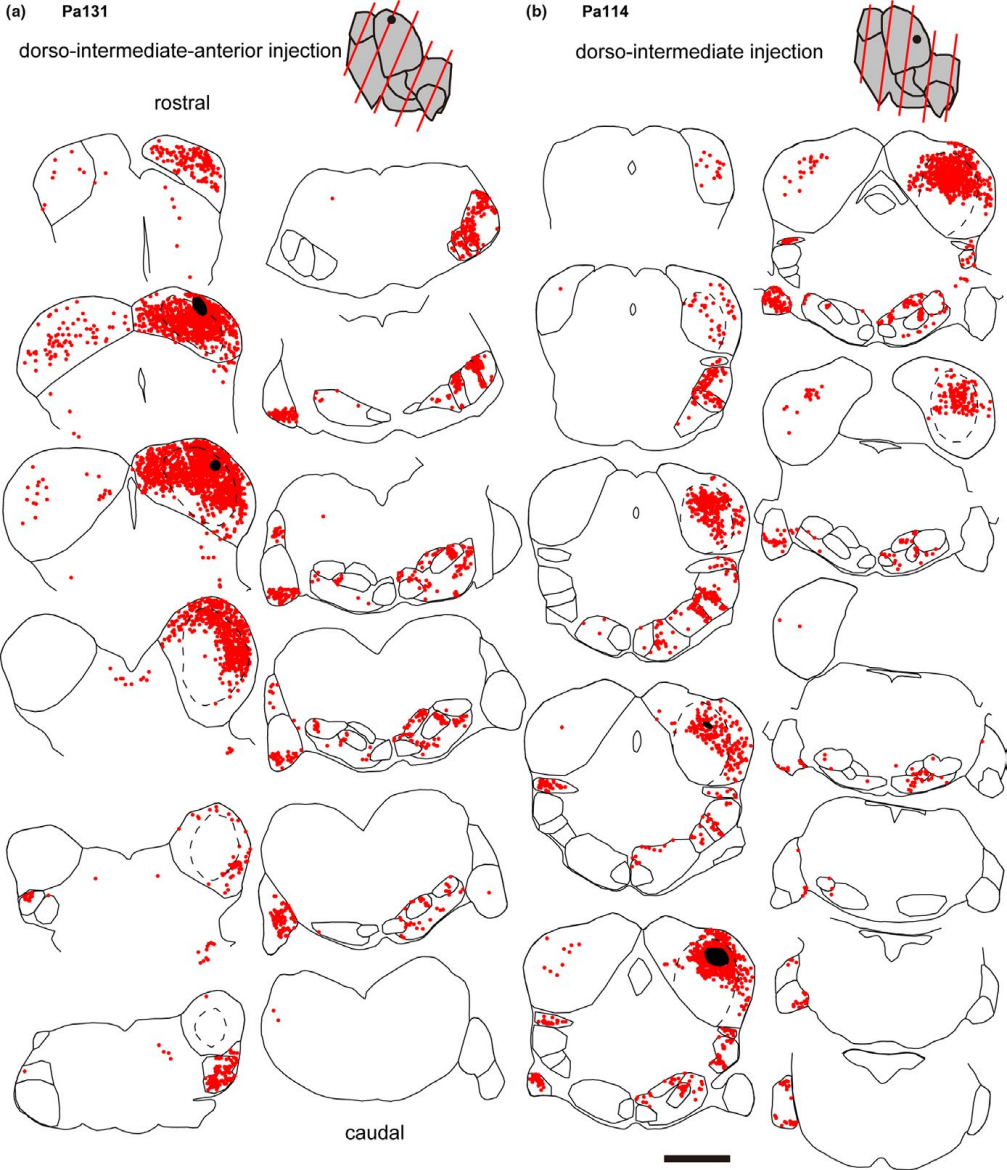
Line drawing showing the traces of auditory nuclei and distribution of labeled cell bodies (red dots) of dorso‐intermediate injection cases, Pa131 (a) and Pa114 (b). See legend of Figure 3 for details. Note that the angle of sectioning was more oblique in Pa131 than other cases

**Figure 5:**
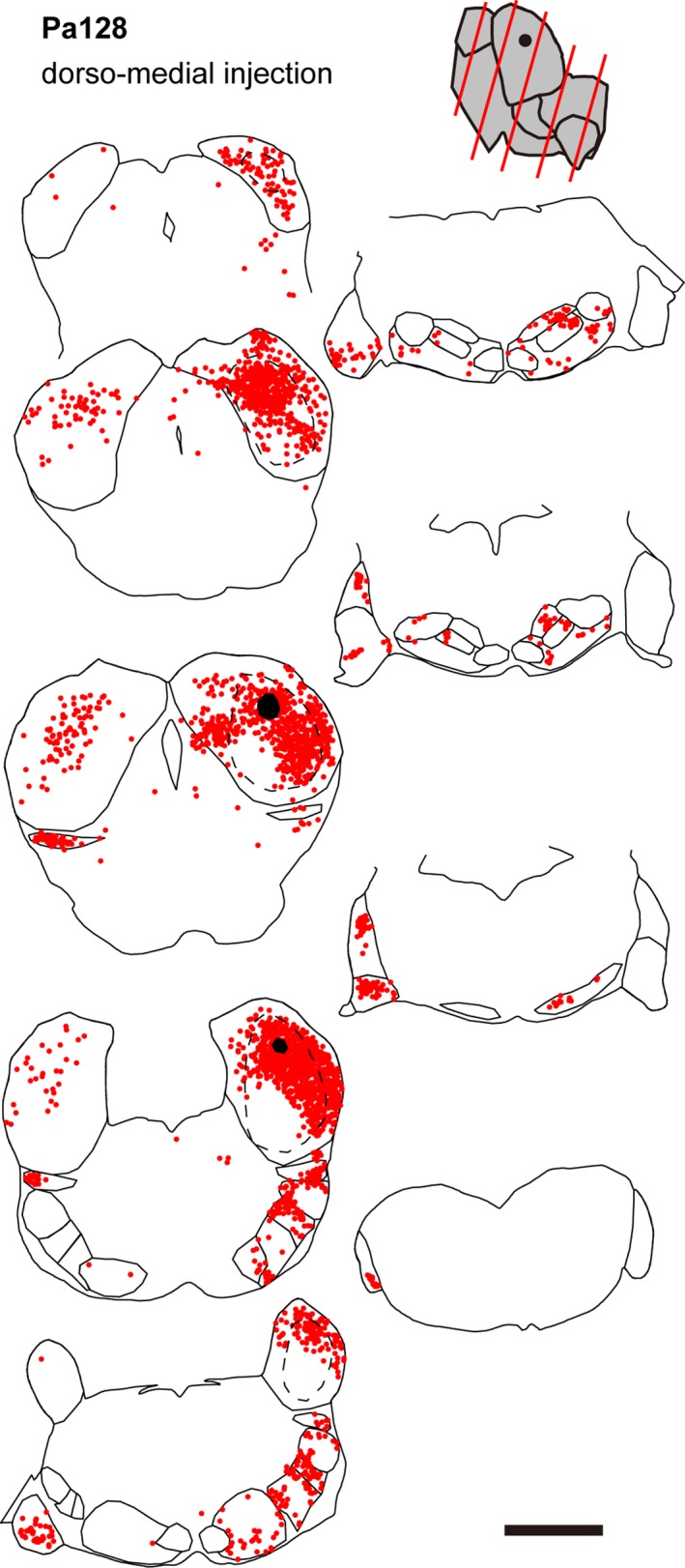
Line drawing showing the traces of auditory nuclei and distribution of labeled cell bodies (red dots) of a dorso‐medial injection case, Pa128. See legend of Figure 3 for details

FG+ cells were found outside the ipsilateral IC (Figures 2, 3, 4, 5). We counted the number of labeled cells in each auditory brainstem nucleus. The total number of FG+ neurons outside the ipsilateral IC was 3,590, 1,936, 2,134, 860, and 1,684 in case Pa119, Pa125, Pa131, Pa114, and Pa128, respectively.

A few cells were found in a nearly symmetrical manner in the contralateral IC (Figures 3, 4, 5). More labeled cells were found in the medial injection case (Pa128). In the dorsal nucleus of the lateral lemniscus (DLL), FG+ cells were found bilaterally (Figures 2a and 3, 4, 5). In the large injection case (Pa119) (Figure 3a), FG+ cells were distributed throughout the nucleus, while the other dorsal injection cases, FG+ cells were distributed in the lateral part of the nucleus (Figures 3, 4, 5). In monaural nuclei of the lateral lemniscus, i.e., ILL, VLLc, and VLLm, labeling was found only in the ipsilateral side (Figures 2b and 3, 4, 5). In Pa119, FG+ cells were found mainly in the center of the nuclei (Figure 3a), while in the other cases, FG+ cells were found in the periphery of the nuclei (Figures 3, 4, 5).

In the SOC, labeled cells were found bilaterally. Inside the SOC (Figure 2c), FG+ cells were densely found in the superior paraolivary nucleus (SPO), MSO, and lateral superior olive (LSO). Fewer cells were found in the periolivary region (PO), which surrounds LSO and MSO and is identified with sparser cell density in Nissl stained sections. In the ipsilateral medial nucleus of the trapezoid body (MTB), few cells located close to the boundary of the nucleus were labeled. In the SPO, FG+ cells found mainly in the ipsilateral side. In the dorsal injection cases, labeled cells were tended to locate in the lateral part, while in the large injection case, FG+ cells were distributed throughout the nucleus. In the MSO, labeled cells were found bilaterally, although the ipsilateral projection seemed stronger. In the dorsal injection cases, labeled neurons were found in dorso‐lateral part of the nucleus, while in the large injection case, more neurons were found in ventro‐medial part. In the LSO, labeled cells were found bilaterally. In the dorsal injection cases, labeled cells were restricted in the lateral part of the LSO, while in the large ventral injection case, more neurons were found medially.

In the CN, we found FG+ cells in all subnuclei, i.e., anteroventral, posteroventral, and dorsal nuclei (AVCN, PVCN, and DCN) in the contralateral side (Figure 2d). In the ventral nuclei (VCN), labeled cells were found in ventral part when the injection was made dorsally, and were sparsely distributed throughout the nuclei in the large ventral injection case (Pa119). In the DCN, topographic organization was clearly evident in the caudal end of the nucleus: in the dorsal injection cases, especially Pa125 and Pa128 (Figures 3b and 5), FG+ cells were found in ventral part, while in the ventral large injection case (Pa119, Figure 3a), FG+ cells were found in dorsal part of the nucleus.

**Figure 6:**
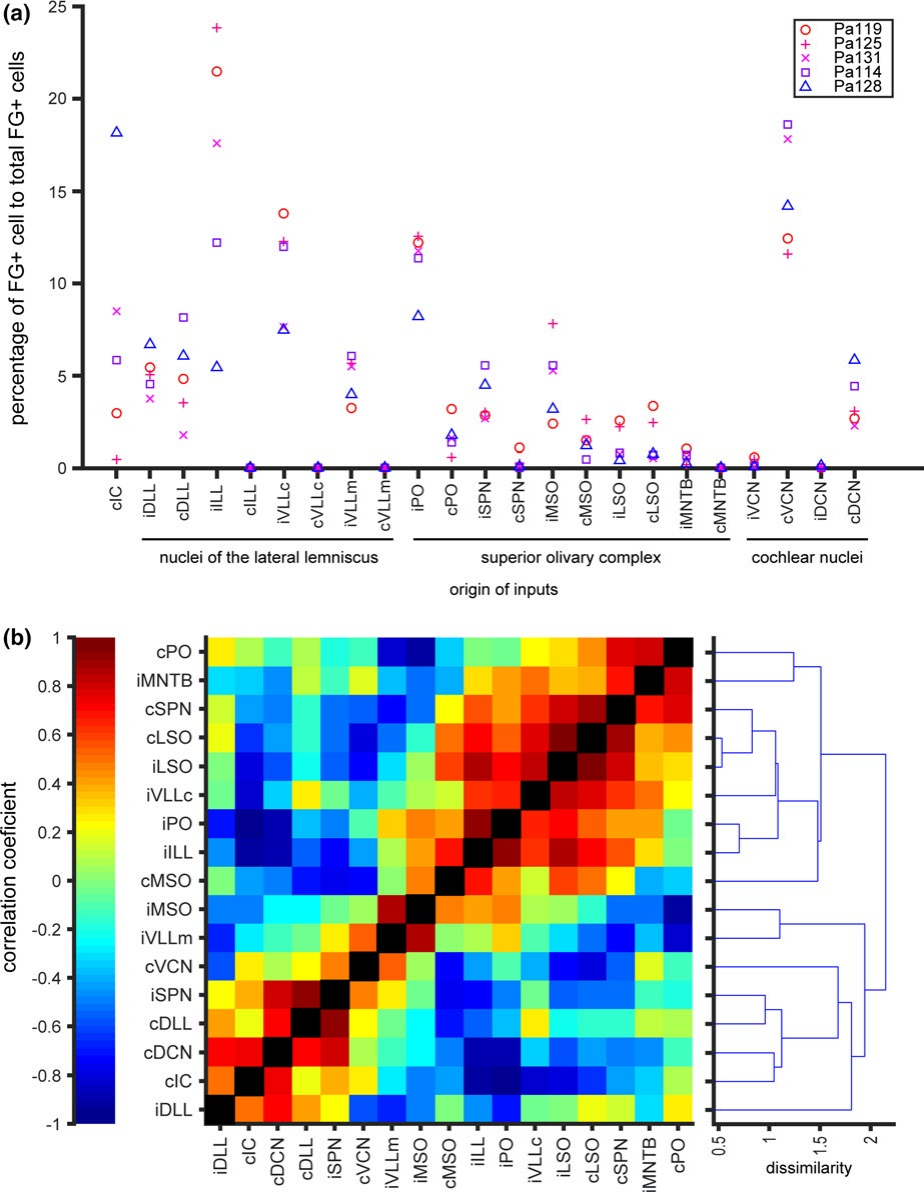
The distribution pattern of retrogradely labeled cells in auditory brainstem nuclei is related to the injection sites. (a) The percentages of FG+ cells in each nuclei to total FG+ cells of individual cases. (b) A heat map showing correlation coefficients of percentages shown in (a) between brainstem nuclei. Nuclei which did not contain FG+ cells in all cases were omitted from the analysis. A dendrogram of dissimilarity of correlation coefficients is shown in right

From the traces of auditory nuclei and plot of FG+ cells outside the ipsilateral IC, we calculated the percentages of labeled cells in each nucleus to total retrogradely labeled cells outside the ipsilateral IC (Figure 6a). Although the sample number was small, we found a tendency that the percentages of FG+ cells are likely to be related to the injection site: The behavior of percentages of nuclei tended to be opposite between the medial injection case (Pa128, triangles) and lateral injection cases (Pa119 and Pa125, circles and “+” in Figure 6a, respectively) in many nuclei. To quantify the relationship between injection sites and percentages of FG+ cells, we calculated correlation coefficients between pairs of nuclei that contained retrogradely labeled cells, created a dendrogram based on the dissimilarity of the correlation coefficients, and a heat map showing clusters of nuclei that are positively correlated (Figure 6b). In general, we found two clusters of correlated nuclei; one mainly consisted of SOC nuclei, and another one consisted of CN and DLL. The result suggests that the composition of brainstem inputs to the ICC is related to the location inside the ICC.

### 3.3 Distribution of FG+ cells inside the ipsilateral IC

**Figure 7:**
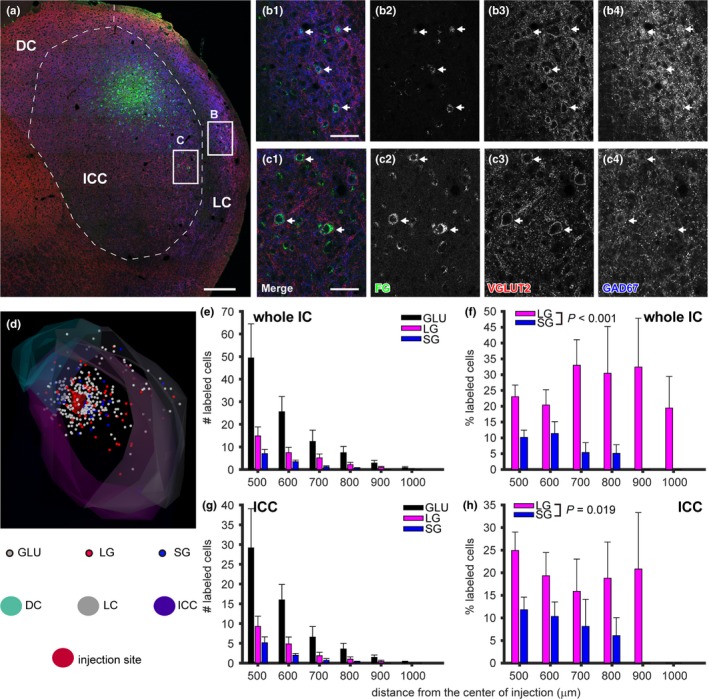
Distribution of retrogradely labeled putative glutamatergic (GLU), large GABAergic (LG), and small GABAergic (SG) neurons in the ipsilateral IC. (a) A montage of tiled micrographs of the IC. Case Pa125. Green, FG; red, VGLUT2; blue, GAD67. (b) A higher magnification image of a box located in the LC of panel (a). Arrows indicate FG+ LG cells. (c) A higher magnification image of a box located in the ICC of panel (a). Arrows indicate FG+ LG cells. Scale bars: 200 μm (a) and 40 μm (b, c). (d) A three‐dimensional reconstruction of IC that received injection of FG. Case Pa6. White, red, and blue dots represent GLU, LG, and SG cells, respectively. Red area indicates injection site. (e–h) Quantitative analysis of spatial distribution of retrogradely labeled cells in the ipsilateral IC of 7 cases. Distance >400 μm is shown to avoid contamination of neurons which directly uptook FG. (e) A histogram of the number of labeled cells in the IC to distance from the center of injection. Bin width was 100 μm. (f) Percentage of LG and SG to all labeled cells in each bin of (e). In the distant part, the percentage of LG increased, while that of SG decreased, and the distribution of LG and SG cells were significantly different (*p* < 0.001, two‐way ANOVA,* N* = 7). (g) Same analysis as (e) was made for ICC cells. (h) Percentage of LG and SG to all labeled cells in each bin of (g). In the distant part, the percentage of LG increased, while that of SG decreased, and the distribution of LG and SG cells were significantly different (*p* = 0.019, two‐way ANOVA,* N* = 7). Error bars indicate standard error of mean

In the ipsilateral IC, FG+ cells were found not only around the injection site but also in the distant part of the IC, including the dorsal cortex (DC) and lateral cortex (LC) of the IC (Figure 7a–c). Under fluorescent micrographs, LG neurons were distinguished from SG neurons by the presence perisomatic rings of VGLUT2+ terminals (arrows in Figure 7b,c). FG+ cells without GAD67 immunoreactivity were regarded as GLU cells since almost all GAD67‐immunonegative cells express VGLUT2 mRNA in mice (Fujimoto, Konno, Watanabe, & Jinno, 2017). From 3‐D reconstruction of the ipsilateral IC, distribution of three cell types and injection site was visualized (Figure 7d), distance from the center of injection was calculated for each cell, and histograms of distance larger than 400 μm from the injection center were created for each cell type and for whole IC and ICC (Figure 7e,g).

We next calculated the average of percentage of LG and SG cells to total FG+ cells in each histogram bin across seven cases (Figure 7f,h). Within the distance of 400 μm from the injection center, the percentages of LG and SG cells were similar and around 10% (not shown). On the other hand, in more distal site to the injection center, the percentage of labeled LG cells increased while that of SG cells decreased. Indeed, the relationship between distance bins and percentage was significantly different between SG and LG cells (2 way ANOVA, *p* < 0.001 for whole IC and *p* = 0.019 for ICC; *N* = 7). The result suggested that LG and GLU cells connect different subdivisions as well as distant sites within the same subdivision, and SG cells work for local circuitry.

## 4 DISCUSSION

In this study, we showed the synaptic organization of ascending and local inputs to the IC of Japanese house bat. Distribution of retrogradely labeled cells inside auditory nuclei was related to the dorso‐ventral location of tracer injection, suggesting tonotopic organization in many brainstem auditory nuclei. We also found that composition of brainstem input sources is related to the location inside the ICC. These findings suggest that ICC is further subdivided into small domains which are specialized for certain functions. Furthermore, analysis of local connection revealed that the small domains are interconnected by LG and GLU cells.

### 4.1 Suggested tonotopic organization

Although we did not perform physiological recording to confirm the tonotopicity of the IC, according to the previous study which determined the tonotopic organization of the IC of Japanese house bat (Goto et al., 2010), the injection site of Pa119 was higher frequency region (>20 kHz), and those of the other cases were lower frequency. Based on the tonotopy of the IC and tonotopic organization of other mammalian species including bats, in the following we suggest the tonotopic organization of auditory brainstem nuclei of the Japanese house bat. Note that the number of samples was small and inter‐sample variations, it is difficult to determine the tonotopic organization in some nuclei.

In mustache bat (*Pteronotus parnelli*), which uses echolocation pulses composing of constant frequency followed by FM (CF‐FM), origins of ascending inputs to the IC were described in detail (Zook & Casseday, 1982b). In the study, the authors showed topographical organization of inputs to the IC. From the projection pattern to IC region with identified best frequency, tonotopic organization of brainstem nuclei is suggested, and consistent with unit recording study on cats (Guinan, Norris, & Guinan, 1972; Spirou, May, Wright, & Ryugo, 1993): CN shows ventral‐to‐dorsal frequency gradient. The LSO has a frequency gradient which increases from lateral to medial. The MSO has dorsal‐to‐ventral tonotopicity. Such relationships are also found in Japanese house bat.

In the dorsal injection cases, FG+ cells were mainly found in the periphery of the NLL. However, it has been shown that tonotopic organization of monaural nuclei of the lateral lemniscus in big brown bat (*Epitesicus fuscus*) is complicated (Covey & Casseday, 1991), and it is difficult to conclude about the tonotopic organization in the NLL of Japanese house bat. Finer injection with best frequency identification is necessary to solve the issue.

### 4.2 Projection of MSO

In Japanese house bat, MSO projects to bilateral IC, although contralateral projection is one‐third weaker than ipsilateral one. In other bat species, projection of MSO of mustache and horseshoe bats is virtually restricted to ipsilateral side (Casseday et al., 1988; Zook & Casseday, 1982b). On the other hand, the MSO of Mexican free‐tailed bat (*Tadarida brasiliensis*) sends axons to the IC bilaterally (Grothe et al., 1994). It is possible that the difference reflects the phylogeny, as Mexican free‐tailed bat is phylogenically closer to Japanese house bat than mustache or horseshoe bat. It is also possible that the difference arise from type of echolocation calls as mustache and horseshoe bats uses calls composed of CF‐FM, while free‐tailed and Japanese house bats use FM calls (Fenton, 1995).

The different projection patterns of MSO may also relate to composition of cell types. In the MSO of horseshoe and mustache bats, GABAergic neurons are sparsely present (Vater et al., 1992), while they are virtually absent in Japanese house bat (Ito et al., unpublished observation). Together, in horseshoe and mustache bats, MSO makes both excitatory and inhibitory influence on the IC in the same side, while in Japanese house bat it make excitatory influence on the bilateral IC. Both patterns are very different from common mammalian plan, and imply its relationship to special auditory function of echolocating bats.

### 4.3 Functional considerations

Correlation of the ratio of input nuclei (Figure 6b) suggests that the composition of brainstem inputs to the ICC is related to the location inside the ICC. Therefore, similar to other non‐echolocating mammals (Cant & Benson, 2006; Loftus et al., 2010) and other bat species (Zook & Casseday, 1987), IC of Japanese house bat is likely to be subdivided into small “synaptic domains.” Small domains are likely to integrate specific combination of input nuclei to code de novo information of sound (Loftus et al., 2010).

It is also noted that synaptic domains are interconnected by LG neurons as well as glutamatergic interneurons (Ito & Oliver, 2014). The computation made in each synaptic domain would be modified by the interconnection of LG and GLU cells. Furthermore, information conveyed by interconnecting LG and GLU cells is likely to be different since both cell types receive different combination of extrinsic inputs (Chen, Cheng, Ito, & Song, 2018) and LG cells receive inputs from GLU cells in different synaptic domains (Ito & Oliver, 2014). Based on studies on rodents, LG neurons integrate information from multiple brainstem nuclei, while GLU cells integrate inputs related to their synaptic domains (Chen et al., 2018; Ito & Oliver, 2014; Ito et al., 2015). Therefore, it is suggested that LG cells control the entire IC circuit according to global state of brainstem nuclei, while GLU cells control according to the state of specific pathways. LG cells are shown to send long‐range projection to thalamus to elicit inhibitory response faster than excitation (Peruzzi, Bartlett, Smith, & Oliver, 1997). The long‐range connection inside the IC may also cause fast inhibition, which is important for coding complex information such as duration (Casseday et al., 1994). To understand the roles of local neurons on the synaptic‐domain‐based information coding, cell‐type specific manipulation of local circuit is required.

## CONFLICT OF INTEREST

The authors declare no conflict of interest.

## AUTHOR CONTRIBUTIONS

TI designed the study, and performed statistical analyses. TI, TF, and KH performed experiments. TF, KH, KIK, and SH prepared the animals. All authors analyzed the data, and wrote the paper.
